# Thermal Performance of Masonry Walls Strengthened with Glazed-Hollow-Bead-Modified ECC

**DOI:** 10.3390/ma19194197

**Published:** 2026-09-30

**Authors:** Xintian Yang, Fa Wang, Hui Liu, Tianao Wu, Yixuan Chen, Jie Liu

**Affiliations:** 1 Changchun Institute of Technology, Changchun 130012, China; 2 Siping Urban Housing Construction Service Center, Siping 136001, China

**Keywords:** B-ECC, glazed hollow beads, thermal conductivity, thermal performance, integrated retrofit, energy efficiency

## Abstract

Existing masonry buildings in severe-cold regions face the dual challenges of structural vulnerability and high heating demand. This study develops a glazed-hollow-bead-modified engineered cementitious composite (B-ECC) and evaluates its thermal performance at the material, wall-component, and building levels within an integrated retrofit strategy. At 50% volumetric sand replacement and 1.5% PVA-fiber content, a B-ECC achieved a thermal conductivity of 0.244 W/(m·K) (52% reduction) while retaining a compressive strength of 43.9 MPa. Wall-level finite-element analysis, validated by hot-box experiments (±5% error), showed that 20 mm overlays per face provide balanced thermal improvement. A whole-building case study of a three-story masonry teaching building in Changchun demonstrated that combining B-ECC overlays with insulation, window replacement, and heat-recovery ventilation reduced annual heating demand from 70,685 to 17,229 kWh/a, achieving a 75.6% energy-saving rate. The primary value of a B-ECC lies in enabling a coordinated structural–thermal retrofit.

## 1. Introduction

### 1.1. Research Background and Significance

Many existing public buildings in the severe-cold regions of northern China were constructed before current seismic and energy-efficiency requirements were established. Aging unreinforced masonry commonly lacks continuous tie elements, ring beams, constructional columns, and reliable wall-to-floor connections; cracking, weathering, carbonation, and mortar deterioration further reduce stiffness and the reliability of load transfer. Seismic design and strengthening standards, together with studies of advanced materials for existing masonry, show that effective intervention must address both global stability and local crack and connection deficiencies [1,2,3,4].

The same building stock also has a poorly performing envelope. Uninsulated or inadequately insulated walls, single glazing, roof heat loss, and uncontrolled air infiltration increase winter heating demand and operating cost in severe-cold climates. Energy-efficiency standards and reviews of existing-building retrofit and life-cycle performance identify envelope transmission and building-system inefficiency as major sources of operational energy use and carbon emissions. Structural strengthening and energy retrofit are nevertheless often designed as separate interventions: rigid mortar or reinforced-concrete overlays may add dead load and create thermal bridges, whereas insulation alone cannot restore the load-bearing capacity or control structural cracking. A compatible strengthening material that contributes to structural reliability, thermal resistance, and durability is therefore needed for a coordinated retrofit strategy [5,6,7,8].

### 1.2. State of the Art

The engineered cementitious composite (ECC) is a promising candidate for this purpose. ECC is a fiber-reinforced cementitious composite designed to exhibit tensile strain hardening through the formation of multiple stable fine cracks rather than one dominant crack. A micromechanical design links fiber properties, matrix composition, and interfacial behavior to tensile ductility and crack-width control. The use of supplementary cementitious materials, particularly high-volume fly ash, can further improve workability, material greenness, and selected mechanical properties when the mixture is properly proportioned. Internal curing and the intrinsically tight crack widths of ECC also support the resistance to early-age cracking and carbonation-related deterioration. These characteristics allow a relatively thin overlay to conform to irregular masonry surfaces, bridge existing cracks, transfer shear, accommodate differential deformation, and add less mass than conventional concrete jacketing [9,10,11,12].

Existing masonry-strengthening methods, including FRP laminates, grouting, and cementitious overlays, can improve in-plane or out-of-plane capacities, but their performance depends strongly on the substrate condition and interface quality. FRP systems may be sensitive to fire and debonding, while conventional cementitious or concrete layers generally provide little thermal benefit. Most ECC studies have consequently concentrated on mechanical response, durability, and mixture sustainability; thermal conductivity has rarely been treated as a primary design variable. Thermal studies of lightweight-aggregate concrete and porous cementitious materials show that density, porosity, aggregate replacement, moisture condition, and pore connectivity govern heat transfer. Lightweight porous aggregates can reduce conductivity, but excessive replacement may increase water demand, disturb fiber dispersion, weaken the interfacial transition zone, and reduce strength. The challenge is therefore to design a thermo-mechanical ECC that lowers heat flow while retaining the ductility, strength, and crack control required for masonry strengthening [13,14,15,16,17,18].

Glazed hollow beads provide a practical route to this balance because their low density and predominantly closed-cell structure can introduce thermal resistance without replacing the cementitious strengthening matrix. However, evidence on the coupled effects of bead dosage and fiber content in the ECC remains limited, and the wall-level consequences of using such a material are not established. In particular, few studies connect mixture optimization to the thickness of an ECC strengthening layer, validate the resulting heat-transfer response at the wall scale, and then quantify the contribution of the strengthening system within a whole-building retrofit. These unresolved links motivate a multi-scale investigation that treats structural and thermal performance as coupled design objectives [19,20].

### 1.3. Objective and Scope

This study develops a glazed-hollow-bead-modified engineered cementitious composite (B-ECC) and evaluates it at the material, wall-component, and building levels. At the material level, glazed hollow beads replace part of the quartz sand, while the PVA-fiber content is varied; thermal conductivity, density, compressive strength, splitting tensile strength, and the resulting thermo-mechanical balance are measured. At the component level, a finite-element heat-transfer model examines masonry thicknesses of 120–490 mm and B-ECC strengthening-layer thicknesses of 0–30 mm, and a chamber experiment is used to check the model before selecting a practical layer thickness [21,22,23,24,25,26,27,28,29,30].

At the building level, the optimized B-ECC system is applied to a representative three-story masonry teaching building in Changchun, a severe-cold region. Baseline masonry, conventional strengthening, B-ECC strengthening alone, and an integrated retrofit that combines a B-ECC with external-wall insulation, roof insulation, high-performance windows, and heat-recovery ventilation are compared. Exterior-wall and roof U-values, annual heating demand, energy-saving rate, and long-term carbon reduction are evaluated within a common framework. The objective is to establish a design pathway that links ECC mixture design, masonry strengthening, envelope heat transfer, and building-scale energy retrofit and to clarify the structural and thermal roles that a B-ECC can play in an integrated intervention [31,32,33,34,35,36,37,38,39,40].

## 2. Theoretical Background and Research Methods

### 2.1. Fundamentals of Heat Transfer Through Building Envelopes

Heat transfer through an exterior building envelope comprises three stages: convection at the exterior surface, conduction through the material, and convection at the interior surface. Under steady-state conditions, the heat flow is identical in all three stages.

#### 2.1.1. Thermal Resistance and Overall Heat-Transfer Coefficient

For a multilayer assembly, the thermal resistances of the individual layers are added in series. The overall heat-transfer coefficient U of the envelope is given by

(1) $$K=\frac{1}{R_{i}+\sum_{i=1}^{n}\frac{\delta_{i}}{\lambda_{i}}+R_{e}}$$

where where R_i_ and Re are the interior and exterior surface thermal resistances, respectively, and di and λ_i_ are the thickness and thermal conductivity of layer i, respectively. According to GB 50189-2015 [23], the U-value of the external walls in severe-cold zone C should not exceed 0.38 W/(m^2^·K) and that of roofs should not exceed 0.30 W/(m^2^·K).

#### 2.1.2. Thermal Inertia and Heat-Storage Performance

Under transient heat-transfer conditions, such as diurnal temperature variation, the heat-storage capacity of the material becomes important. The thermal inertia index *D* is used to evaluate heat-storage performance:

(2) *D* = *R* × *S*

where the heat-storage coefficient *S* is

(3) $$s=\sqrt{\lambda \cdot c\cdot \rho }$$

where *c* is the specific heat capacity and *ρ* is the density. Buildings with a high thermal inertia (*D* ≥ 10) can use daytime solar gains to reduce nighttime heating loads under intermittent heating schedules, such as those of schools.

The thermal diffusion time constant is a key measure of the wall’s thermal response rate:

(4) $$\tau =\frac{\rho \cdot c\cdot \delta^{2}}{\pi \cdot \lambda }$$

The thermal diffusion time constant of a homogeneous layer, *τ = ρcδ^2^/(πλ)*, is a measure of its thermal response rate. For the 20 mm B-ECC overlay alone (*ρ* = 1550 kg/m^3^, *c* = 1050 J/(kg·K), λ = 0.244 W/(m·K)), the time constant is τ ≈ 0.86 h, which is much smaller than the 24 h diurnal cycle. This indicates that the B-ECC layer itself responds rapidly to temperature changes and can be treated as a quasi-steady state at the daily-average time scale.

Classification and interpretation of thermal inertia: According to GB 50176-2016, *D* ≥ 10 indicates a high thermal inertia and is suitable for regions with large diurnal temperature ranges; 5 ≤ *D* < 10 indicates a moderate thermal inertia; and *D* < 5 indicates a low thermal inertia. The *D*-value of a 490 mm exterior masonry wall is approximately 9.6, near the upper limit of the moderate range. Strengthening both faces with a B-ECC increases *D* to approximately 10.8, moving the wall into the high-inertia range and improving indoor thermal stability in intermittently heated buildings.

#### 2.1.3. Mean U-Value Accounting for Thermal Bridges

In practice, an exterior wall consists of the main wall area (96–98%) and thermal bridges (2–4%). The mean heat-transfer coefficient Um is calculated according to GB 50176-2016 as

(5) $$K_{m}=K+\frac{\sum \Psi_{i}L_{i}}{A}$$

where *ψ*_i_ is the linear thermal transmittance of thermal bridge *i*, *L*_i_ is its length, and *A* is the total wall area. Thermal-bridge treatment is therefore essential in an energy retrofit.

### 2.2. Basic Characteristics and Thermal Mechanisms of an ECC

#### 2.2.1. Constituents and Microstructure of ECC

An ECC consists of cementitious materials (cement and fly ash), fine aggregate (40–80 mesh quartz sand), PVA fibers (16 mm long, 40 μm in diameter, and 1.5–2.0% by volume), and a polycarboxylate superplasticizer. Compared with conventional concrete, an ECC has an optimized pore structure, a porosity of 15–20% rather than 25–35%, high interfacial strength, and a multiple-fine-cracking mechanism that limits crack widths to less than 100 μm.

#### 2.2.2. Physical Mechanisms Governing Thermal Conductivity

The thermal conductivity of a material is governed by the combined contributions of conduction through the solid phase and through the gas in its pores. For a porous medium, thermal conductivity can be expressed as

(6) $$\lambda =\lambda_{\mathrm{solid}}(1-p)+\lambda_{\mathrm{gas}}\cdot p+\Delta \lambda_{\mathrm{radiation}}$$

where λ_solid_ is the thermal conductivity of the solid phase (approximately 2.1 W/(m·K)), *λ*_gas_ is that of the gas phase (approximately 0.024 W/(m·K)), *p* is the porosity, and *λ*_radiation_ is the high-temperature radiation correction, which can be neglected under the ambient-temperature conditions considered here.

The equation indicates that increasing porosity effectively reduces thermal conductivity. Glazed hollow beads, with a density of 80–120 kg/m^3^, a closed-cell ratio of at least 80%, and a thermal conductivity of 0.042 W/(m·K), were incorporated into an ECC by volumetrically replacing quartz sand. At a replacement ratio α of 50%, the density decreased from approximately 2000 to 1500 kg/m^3^ and the porosity increased from 15% to approximately 35–40%.

Correction for closed porosity: The glazed hollow beads have a closed-cell ratio of 93%, indicating that the pores inside the beads are essentially sealed. The effective porosity, p_eff_, participating in gaseous conduction, should therefore exclude the contribution of closed pores from the total porosity:

(7) *P*_eff_ = *P*_total_ × (1-closed-cell ratio) ≈ (35–40%) × 7% ≈ 2.5–2.8%

This correction explains the nonlinear decrease in B-ECC thermal conductivity from 0.51 to 0.244 W/(m·K): the closed-cell structure suppresses gas convection, producing a greater increase in thermal resistance than would be expected solely from the increase in total porosity.

Thermophysical comparison with conventional insulation materials: Based on their heat-transfer mechanisms, materials can be divided into three categories: conventional concrete (*λ* = 2.0 W/(m·K), solid-phase dominated), a B-ECC (*λ* = 0.244 W/(m·K), mixed solid–gas conduction), and insulation materials such as EPS (*λ* = 0.032 W/(m·K), gas-phase dominated). A B-ECC occupies a transition zone between structural and insulation materials and combines characteristics of both.

#### 2.2.3. Mixture Design Strategy for Balancing Mechanical and Thermal Performance

This section discusses the mixture design strategy for balancing thermal and mechanical performance, rather than presenting a fully coupled thermo-mechanical analysis. The central question is whether the incorporation of glazed hollow beads substantially impairs the mechanical strength of an ECC. By controlling the water-to-binder ratio, supplementary cementitious material content, and superplasticizer dosage, an optimum balance can be achieved between thermal and mechanical performances. This tunability is a core advantage of the ECC: appropriate mixture design can coordinate both properties rather than impose a simple trade-off. The theoretical basis for the trade-off between thermal-conductivity reduction and strength retention is explained through the pore structure and bead-to-bead contact mechanisms discussed in Section 2.2.2.

### 2.3. Heat-Transfer Model of the Composite Wall

#### 2.3.1. Development of the Finite-Element Thermal Model

Steady-state heat conduction was analyzed using ABAQUS(2023). The governing heat-conduction equation is

(8) $$\frac{\partial }{\partial x}(\lambda_{x}\frac{\partial T}{\partial_{x}})+\frac{\partial }{\partial y}(\lambda_{y}\frac{\partial T}{\partial y})+Q_{internal}=0$$

Under steady-state conditions ($\partial T/\partial_{t}=0$), the exterior surface was maintained at −20 °C, the interior surface at 18 °C, and the lateral boundaries were adiabatic. The steady-state temperature field was solved to obtain the heat flow *Q* through the wall and, subsequently, its overall heat-transfer coefficient U.

Explanation of the applicability of the steady-state assumption: This study employs a finite-element model to calculate the daily average heat-transfer coefficient for composite walls under winter heating conditions. According to the principles of harmonic analysis of dynamic thermal characteristics specified in ISO 13786:2017 [41], the wall heat transfer under periodic external disturbances can be decomposed into the sum of a steady-state component corresponding to the average temperature and a harmonic component corresponding to the temperature fluctuations. For the composite masonry-B-ECC wall assembly considered here (masonry thickness of 240–490 mm), the thermal time constant τ ranges from approximately 4 to 8 h. While this is not negligible compared to the 24 h diurnal period, it is sufficiently less than the 24 h that the steady-state component of the heat flux dominates the daily average, and the harmonic component contributes primarily to intra-day fluctuations around the mean. Therefore, the steady-state analysis provides a reasonable estimate of the daily average heat-transfer coefficient, which is the quantity of interest for comparing retrofit scenarios. However, instantaneous heat flux variations induced by diurnal temperature fluctuations are not captured by this model [41].

#### 2.3.2. Framework for Parametric Sensitivity Analysis

The effects of the following variables on the U-value were systematically evaluated: masonry thickness *δ*_wall_ (120, 240, 370, and 490 mm), ECC strengthening-layer thickness *δ*_ECC_ (0, 10, 20, and 30 mm), and strengthening material (none, an ECC, or reinforced concrete). An orthogonal design was used to generate the analysis cases and derive the relationship U = f (*δ*_wall_, *δ*_ECC_).

#### 2.3.3. Method for Calculating Building Energy Consumption

Building heating demand was calculated as the sum of transmission, ventilation, and infiltration heat losses. Dynamic hourly calculations were conducted using the BECS(2024) building energy-efficiency design software based on the monthly quasi-dynamic method of ISO 13790 [42]. Seasonal and orientation-dependent solar radiation, building heat storage, intermittent heating schedules, and three-dimensional heat-flow effects were considered. The main model inputs were as follows: weather data for the Changchun severe-cold zone (CSWD typical meteorological year); a daytime occupancy schedule from 8:00 to 17:00 on weekdays with an internal gain of 4 W/m^2^ for occupants, lighting, and equipment combined; an infiltration rate of 0.5 air changes per hour (ach) based on Class II airtightness; an intermittent heating schedule from 8:00 to 22:00 daily during the heating season (October 20 to April 10, totaling 169 days); and a ventilation rate of 1.0 ach when natural ventilation was considered. The entire heating season was selected as the simulation period. The baseline model (Scenario 1) was calibrated against the actual annual heating bills of the case building (approximately RMB 21,000/year, equivalent to 70,685 kWh/year), and the simulated heating demand showed a deviation of less than 5%, confirming the validity of the baseline model.

### 2.4. Theoretical Framework for the Thermal Performance of an ECC

#### 2.4.1. Effect of Pore Structure on Thermal Conductivity

The thermal conductivity of a porous material is governed by four heat-transfer mechanisms: sensible conduction through connected solid paths, which is the primary contribution; gas conduction through molecular collisions within pores; radiative transfer across pore walls at high temperatures, which is negligible under ambient conditions; and convective transfer caused by gas movement in open pores. In a B-ECC, the high closed-cell ratio of the glazed hollow beads (93%) effectively suppresses the latter two mechanisms. Thermal resistance therefore arises mainly from the elongation of solid conduction paths and the introduction of gaseous conduction paths.

#### 2.4.2. Effect of Fibers on Thermal Performance

The thermal conductivity of PVA fibers is approximately 0.08–0.12 W/(m·K), substantially lower than the 2.1 W/(m·K) of the cementitious matrix. Incorporating fibers therefore introduces low-conductivity inclusions into the matrix. Increasing the fiber content from 1.5% to 2.0% produced an additional thermal-conductivity reduction of approximately 5–8%. The randomly distributed fiber network also increases the tortuosity of the heat-flow path and thereby extends the effective conduction path.

#### 2.4.3. Theoretical Basis for Incorporating Glazed Hollow Beads

The effect of glazed hollow beads on thermal performance can be divided into two stages. At replacement ratios of 0–50%, the beads are fully coated by cement paste and thermal conductivity decreases approximately linearly, primarily because of increasing porosity. At replacement ratios of 50–100%, direct bead-to-bead contact begins, the marginal reduction in thermal conductivity diminishes, and mechanical performance deteriorates more rapidly as beads fracture under compression. This two-stage behavior provides a theoretical basis for determining the optimum replacement ratio.

## 3. Experimental Study of the Thermal Performance of B-ECC

### 3.1. Experimental Design and Material Preparation

#### 3.1.1. Raw Materials and Their Properties

**Figure 1:**
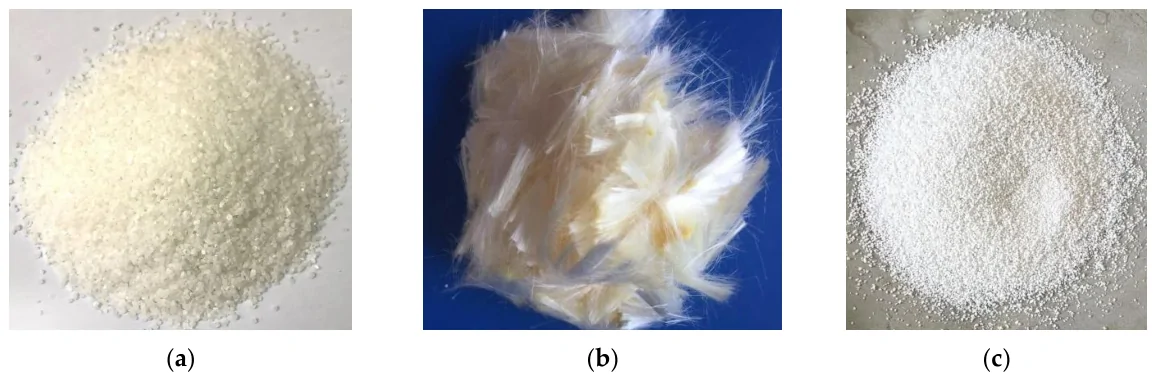
Raw materials used in the experiments: (**a**) 40–80 mesh quartz sand; (**b**) PVA fibers; and (**c**) 50–80 mesh glazed hollow beads.

The composite comprised cementitious materials, fine aggregate, water, admixture, and reinforcing fibers, with constituent types and proportions selected for the intended service conditions. P.O 42.5R ordinary Portland cement was used, and its basic physical and mechanical properties complied with Common Portland cement (GB 175-2023) [21]. Grade I fly ash was selected because of its relatively high reactivity and ability to participate in cement hydration. Quartz sand with a particle size of 40–80 mesh was used; it met the requirements for high-performance cementitious composites and complied with JGJ 52 [22]. Glazed hollow beads volumetrically replaced the quartz sand. A replacement ratio of 100% indicates that the entire volume of fine aggregate consisted of glazed hollow beads, with no quartz sand present in the mixture. To isolate the effect of aggregate type, the glazed hollow beads were sieved to the 40–80 mesh range to match the particle size of the quartz sand. Sieve analysis confirmed that the particle size distributions of the two aggregates were comparable, with over 90% of both materials falling within the 40–80 mesh range. Their principal properties are listed in Table 1. The reinforcing material was domestically produced PVA fiber, 16 mm in length, whose properties are listed in Table 2. The raw materials are shown in Figure 1.

#### 3.1.2. Selection and Optimization of the Reference Mixture

The mixture was designed to achieve a 28-day compressive strength of at least 40 MPa, uniform fiber dispersion, and satisfactory workability. Fiber dosage was determined using the fiber-content equation, and glazed hollow beads volumetrically replaced quartz sand. For clarity, the term “reference mixture” as used here refers to the recommended/optimized mixture rather than a null-control mixture. A separate control mixture (0% fiber content and 0% glazed-hollow-bead replacement) was also prepared for comparison; its composition was identical to that shown in Table 3, except that the quartz sand content was 677.0 kg/m^3^, the glazed-hollow-bead content was 0 kg/m^3^, and no PVA fibers were added.

During fiber-content screening, specimens containing fiber volume fractions of 2.0% and 1.5% were prepared and tested for compressive strength at 3, 7, and 28 days. Both mixtures met the target of at least 40 MPa. The 2.0% mixture was prone to fiber agglomeration during mixing, whereas the 1.5% mixture exhibited better dispersion without the loss of strength. A fiber content of 1.5% was therefore selected for the subsequent investigation.

### 3.2. Cube Compressive-Strength Test

**Figure 2:**
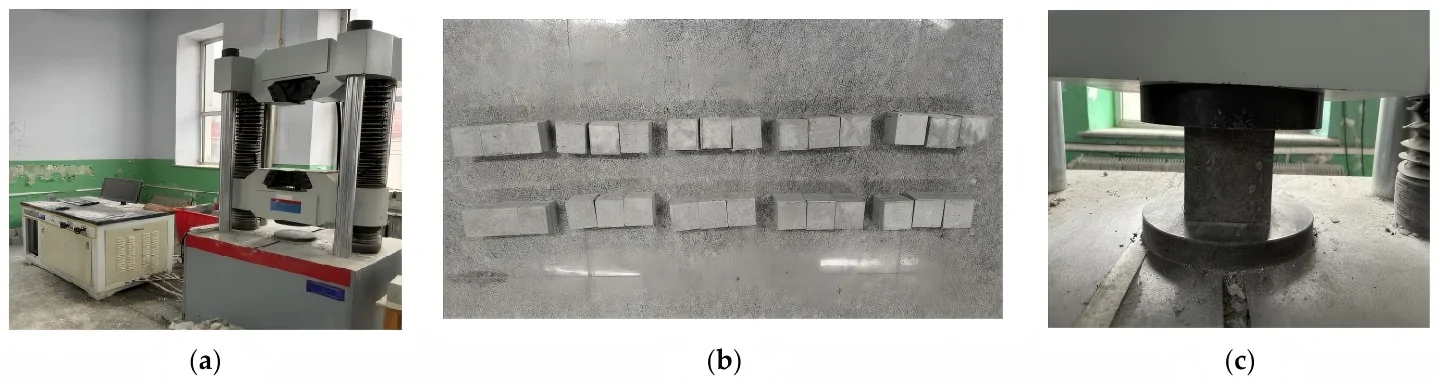
Cubic compressive-strength test: (**a**) YAW-2000 compression-testing machine; (**b**) test specimen fabrication; and (**c**) test specimen fabrication.

The tests were conducted on the YAW-2000 (Changchun Keda Testing Machine Manufacturing Co., Ltd., Changchun, China) microcomputer-controlled electro-hydraulic servo hydraulic compression testing machine in accordance with the “Standard for Test Methods for Physical and Mechanical Properties of Concrete” [1] (GB/T 50081–2019). The test specimens were cubic specimens with dimensions of 100 × 100 × 100 mm; each group consisted of three specimens. All specimens were to be clean and well-organized prior to testing, with dimensional deviation not exceeding 2%. The equipment employed a load-controlled loading method at a rate of 0.3 MPa/s, applying continuous, uniform loading until specimen failure; the load–displacement curve was recorded. All test specimens were cured in a standard curing room for 28 days; the compressive strength was calculated and is presented in Figure 2.

The calculation of concrete cube compressive strength is given by Equation (9):

(9) $$f_{\mathrm{cc}}=\alpha \frac{F}{A}$$

*F*—Test specimen failure load, N.

*A*—Test specimen pressure-bearing area, mm^2^.

*α*—Dimensional conversion factor, 100 mm × 100 mm × 100 mm. The specimen is a non-standard specimen; the dimensional conversion factor is taken as 0.95.

*f*_cc_—Concrete cube specimen compressive strength, MPa; calculation result is accurate to 0.1 MPa.

### 3.3. Splitting Tensile Test

**Figure 3:**
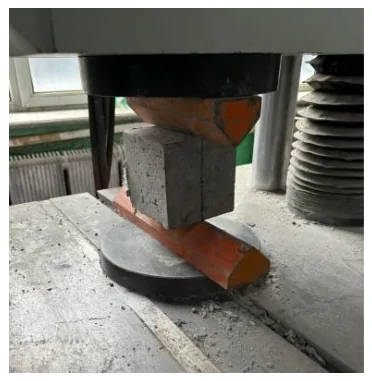
Split tensile test.

The test was conducted on the YAW-2000 (Changchun Keda Testing Machine Manufacturing Co., Ltd., Changchun, China). experimental machine; triangular iron spacers were placed above and below the specimen and a continuous, uniform loading was applied at a rate of 0.03 MPa/s until failure occurred, from which the splitting tensile strength was calculated, as shown in Figure 3.

According to the “Standard for Test Methods for Physical and Mechanical Properties of Concrete” (GB/T 50081-2019) [1], the calculation of concrete tensile splitting strength is given by Equation (10):

(10) $$f_{\mathrm{ts}}=\alpha \frac{2F}{\pi A}$$

*α*—Dimensional conversion factor; the 100 mm × 100 mm × 100 mm specimen is a non-standard specimen and can be set to 0.85;

*f*_ts_—Concrete splitting tensile strength, MPa; the calculation results are accurate to 0.1 MPa.

### 3.4. Thermal-Conductivity Measurement

**Figure 4:**
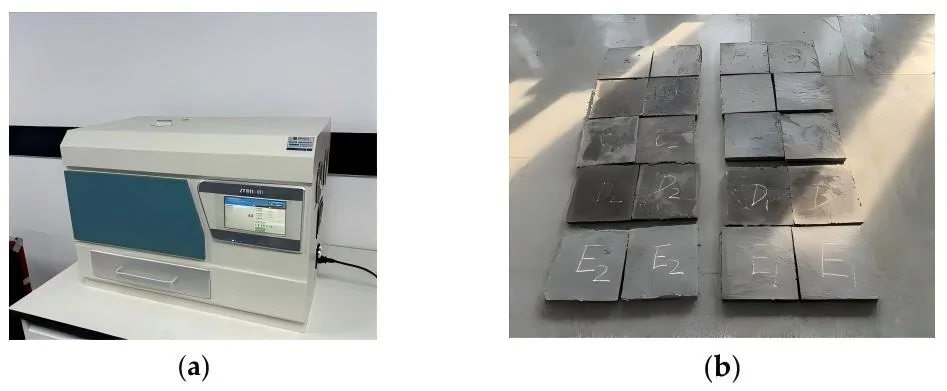
Thermal-conductivity determination test: (**a**) JTRG-III Thermal Conductivity Tester; (**b**) insulation specimens.

This test was conducted in accordance with the standard “Determination of Steady-State Thermal Resistance and Related Properties of Adiabatic Materials—Protective Thermal Plate Method” (GB/T 10294-2008) [43] for specimen preparation. The thermal conductivity was measured using a JTRG-III Thermal Conductivity Tester (Xi’an Xiatech Electronic Technology Co., Ltd., Xi’an, China); the test ambient temperature was maintained at 20 ± 2 °C and the relative humidity at 60 ± 5% RH. All parameters measured by the instrument complied with the specified requirements. This testing instrument is a single-specimen thermal-conductivity tester; specimens with dimensions of 300 mm × 300 mm × 30 mm were prepared, with three specimens per set. The specimens were cured in a standard curing room for 28 days; upon completion of curing, they were oven-dried until reaching a constant weight, then removed and allowed to cool to room temperature before the thermal-conductivity measurement was performed. To ensure the accuracy of the thermal-conductivity determination, a replicate test was commissioned to the Jilin Provincial Construction Engineering Quality Inspection Center. For this replicate test, a dual-specimen thermal-conductivity tester was employed; two specimens per set were used for measurement, and all specimens had identical dimensions, as shown in Figure 4.

The thermal-conductivity calculation formula is

(11) $$\lambda =\frac{\Phi •d}{A(T_{1}-T_{2})}$$

where

Φ—Average heating power of the heating unit’s metering section, *W*;

*T*_1_—Average hot surface temperature of the specimen, *K*;

*T*_2_—Average cold surface temperature of the specimen, *K*;

*d*—Specimen average thickness, m.

### 3.5. Effect of Glazed-Hollow-Bead Content on Thermal Performance

#### 3.5.1. Experimental Design and Test Matrix

Ten mixtures were prepared by varying the volumetric replacement ratio of glazed hollow beads in the reference mixture. Five replacement levels, from 0% to 100% in increments of 25%, were combined with two fiber contents, 2.0% and 1.5%.

When the fiber content was reduced from 2.0% to 1.5%, the 0.5% reduction in fiber volume was compensated by quartz sand or glazed hollow beads to maintain a constant total volume. At a given replacement ratio, the absolute quantities of quartz sand and glazed hollow beads therefore differed slightly between fiber contents. At a 50% replacement ratio, for example, the quartz-sand dosage was 338.5 kg/m^3^ for both the 2.0% and 1.5% mixtures after rounding, because the influence of the small change in fiber volume on aggregate dosage was less than 1%.

A staged fiber-addition procedure was used. The dry powders were mixed for 2 min, water and superplasticizer were added and mixed for 5 min, and the mixture was transferred to a horizontal drum mixer. Fibers were added in three to four batches, with 3 min of mixing after each addition. The total mixing time was 15–20 min, until the fibers were uniformly dispersed.

The compositions of all ten tested mixtures—combining five glazed-hollow-bead replacement ratios (0%, 25%, 50%, 75%, and 100%) with two PVA-fiber contents (1.5% and 2.0%)—were derived from the recommended mixture in Table 3 by adjusting the quartz sand and glazed-hollow-bead dosages proportionally, according to the replacement ratio, and compensating for the small volume change associated with the fiber content difference. At each replacement ratio, the total fine aggregate volume was held constant; the mass of quartz sand replaced by glazed hollow beads was calculated based on their respective densities (1600 kg/m^3^ for quartz sand and 110 kg/m^3^ for glazed hollow beads). All other constituents (cement, fly ash, water, and superplasticizer) were kept constant across all mixtures.

#### 3.5.2. Measurement and Analysis of Thermal Conductivity

Thermal conductivity was measured by the steady-state heat-flow-meter method in accordance with GB/T 10294. A JTRG-III Thermal Conductivity Tester (Xi’an Xiatech Electronic Technology Co., Ltd., Xi’an, China) heat-flow meter with an accuracy of ±3% was used. Cylindrical specimens, 100 mm in diameter and 15–30 mm thick, were dried to constant mass and tested at a mean temperature of 25 ± 2 °C, with a temperature difference of 10 °C.

**Figure 5:**
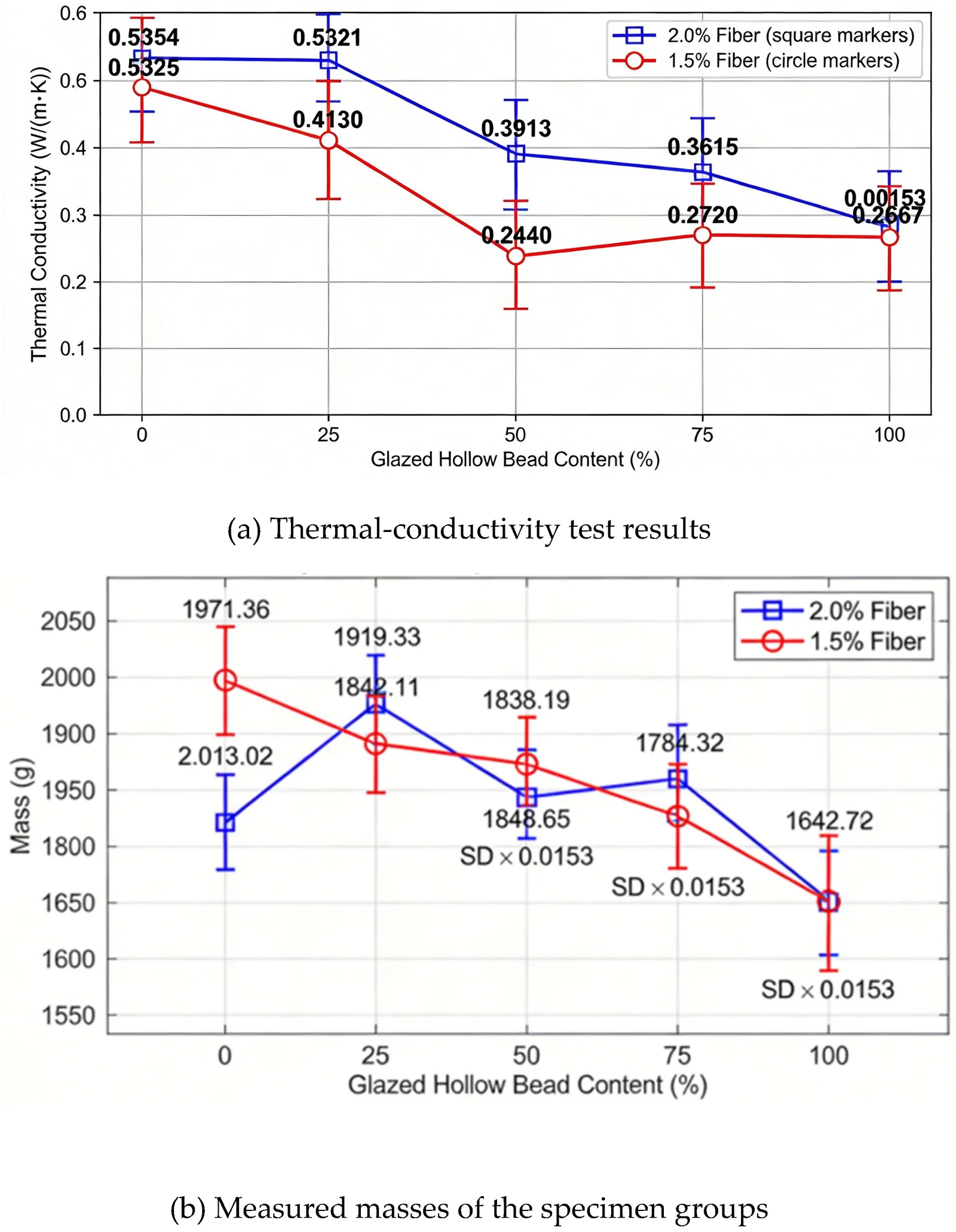
Thermal-conductivity test results.

As shown in Figure 5a, thermal conductivity decreased with increasing replacement ratio. For fiber contents of 2.0% and 1.5%, respectively, it decreased from 0.510 to 0.350 W/(m·K), a reduction of 31.4%, and from 0.510 to 0.380 W/(m·K), a reduction of 25.5%. At the same replacement ratio, the higher fiber content produced the lower thermal conductivity because the thermal conductivity of PVA fibers (approximately 0.08–0.12 W/(m·K)) was substantially lower than that of the cementitious matrix. A mixture with 1.5% fiber and 50% glazed-hollow-bead replacement is recommended. It exhibited a thermal conductivity of 0.244 W/(m·K), which was 52% lower than that of the original masonry; a 28-day compressive strength of 43.9 MPa; a 28-day splitting tensile strength of 3.2 MPa; and a density of approximately 1550 kg/m^3^, representing a 25% reduction, as shown in Figure 5b.

#### 3.5.3. Coupled Evaluation of Mechanical Performance

**Figure 6:**
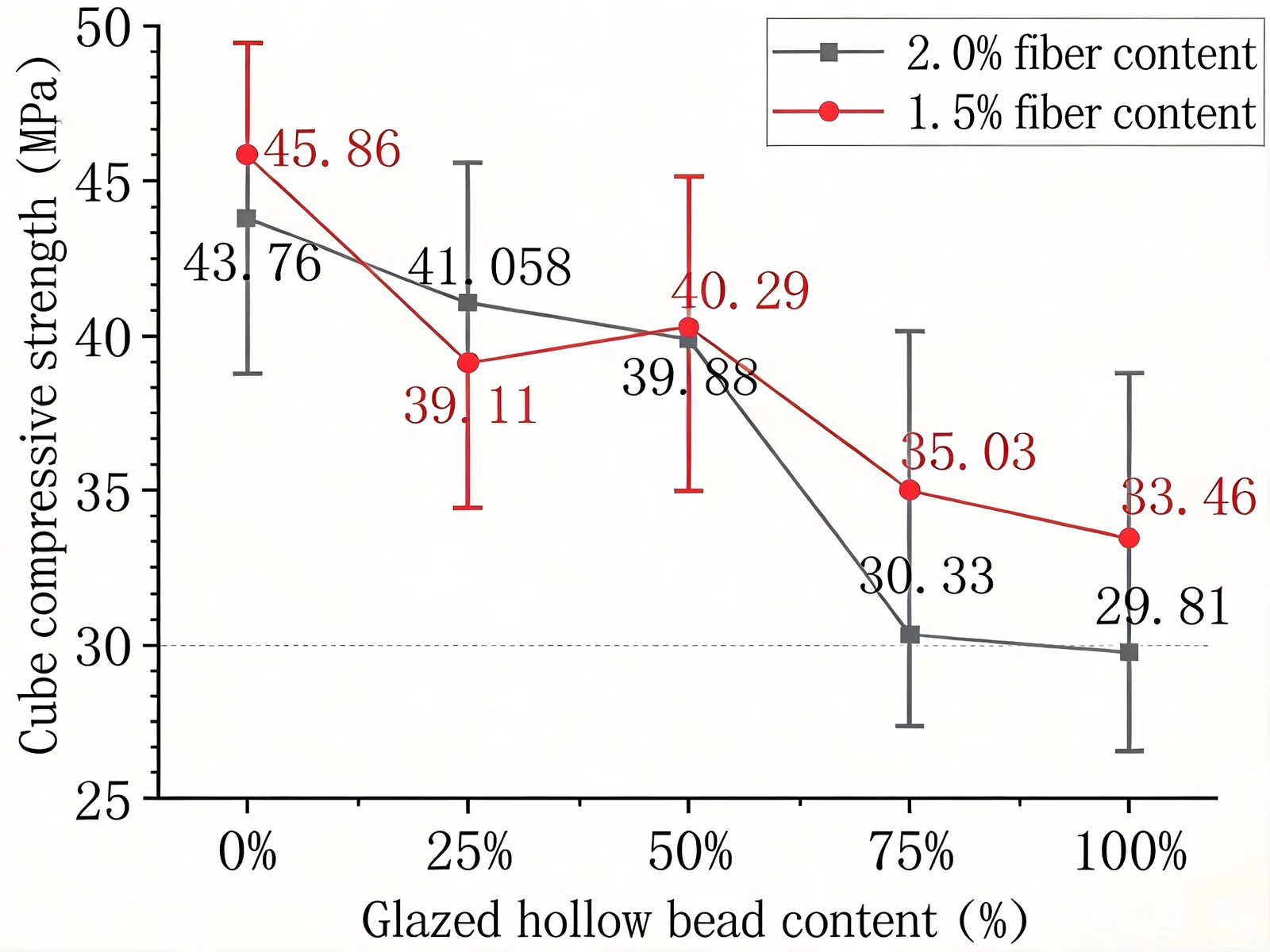
Cube compressive-strength test results.

At both fiber contents, the 28-day compressive strength decreased as the glazed-hollow-bead content increased. As shown in Figure 6, the strength of the 2.0% fiber mixture decreased markedly when the volumetric replacement ratio increased from 50% to 75%, whereas the strength of the 1.5% mixture first increased slightly and then decreased. Across replacement ratios of 0–100%, the strength losses were 31.8% and 27.0% for the 2.0% and 1.5% fiber mixtures, respectively. At 50% replacement, the loss in compressive performance was small and the retained strength remained high. At low replacement ratios, the beads were fully coated by high-strength cement paste and the mortar fraction carried much of the load. Above 50% replacement, bead-to-bead contact increased and the beads could fracture under compression.

#### 3.5.4. Theoretical Interpretation of Thermo-Mechanical Coupling

To quantify the overall performance of each mixture, a combined thermo-mechanical performance index *η* was defined as

(12) $$\eta =\frac{strength\;retention\;ratio}{thermal\;conductivity\;retention\;ratio}=\frac{f_{cc,\alpha }/f_{cc,0}}{\lambda_{\alpha }/\lambda_{0}}$$

where *f*_cc_, *α* and *λ*_α_ were the compressive strength and thermal conductivity, respectively, at replacement ratio *α* and *f*_cc_, 0 and λ_0_ were the corresponding values of the unmodified reference mixture (α = 0%). The numerator, *f*_cc_, *α*/*f*_cc_, 0, represents the retained mechanical performance. The denominator, *λ*_α_/*λ*_0_, represents the retained (relative) thermal conductivity—i.e., the fraction of the baseline conductivity remaining at replacement ratio *α*—rather than the fractional reduction in conductivity (1 − *λ*_α_/*λ*_0_). A smaller value of *λ*_α_/*λ*_0_ therefore corresponds to a greater reduction in thermal conductivity and a larger thermal improvement. Consequently, an increase in *η* may result from either an improved retention of mechanical strength or a decrease in relative thermal conductivity (i.e., greater thermal improvement) or both.

This normalized ratio-of-retention-ratios index is proposed here as a study-specific descriptive and comparative metric for visualizing the thermo-mechanical trade-off associated with glazed-hollow-bead replacement. No directly equivalent index has been identified in the existing ECC literature, and the resulting *η* values should therefore be interpreted as relative indicators of trend rather than as an absolute, dimensionally validated design criterion.

**Figure 7:**
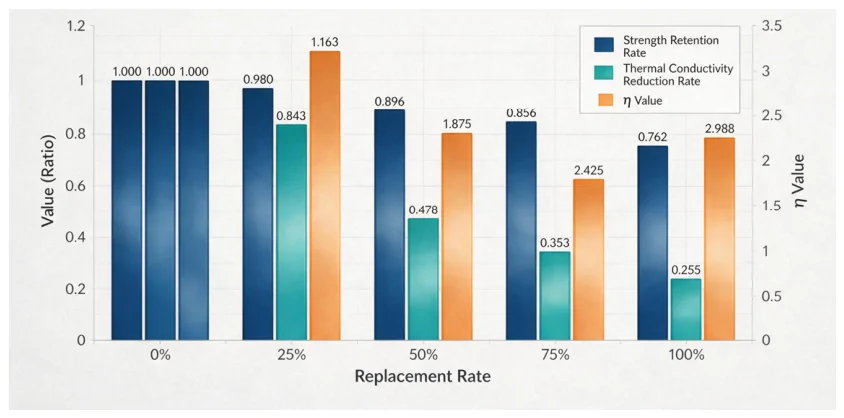
Values of *η* at different replacement ratios.

As shown in Figure 7, *η* increased monotonically with the replacement ratio, from 1.000 at 0% to 2.988 at 100%, and therefore has no interior maximum within the tested range. Consequently, *η* alone cannot be used to identify an optimum replacement ratio through unconstrained mathematical maximization. To resolve this, the selection of the optimum replacement ratio was formally reformulated in Equation (12) as a constrained optimization problem, in which *η*(*α*) is maximized subject to the compressive-strength design requirement established in Section 3.1.2 (*f*_cc_(α) ≥ 40 MPa). Substituting the measured strength values shows that only *α* = 0% and *α* = 50% satisfy this constraint, so that the feasible set is *A* = {0%, 50%}; since *η*(*α*) is monotonically increasing, the solution is *α* = max(*A*) = 50%.

This result is further supported by two complementary observations. First, saturation of the thermal-conductivity improvement: at replacement ratios of 0–50%, the beads are fully coated by cement paste and thermal conductivity decreases approximately linearly; beyond 50%, bead-to-bead contact becomes significant, the marginal reduction in conductivity diminishes sharply, and conductivity even increases slightly at 75–100% replacement (0.244→0.272→0.267 W/(m·K) for the 1.5% fiber series). This indicates that little additional thermal benefit is obtained beyond 50% replacement. Second, diminishing incremental gain in η: although η increases monotonically, the increment between successive replacement levels (Δη = 0.163, 0.712, 0.550, and 0.563 across the four 25% intervals) is largest between 25% and 50% and declines thereafter, indicating that further increases in replacement ratio beyond 50% yield comparatively limited additional benefit to the overall thermo-mechanical trade-off.

Only α = 0% and α = 50% satisfy the strength constraint; the feasible set is therefore *A* = {0%, 50%}. Since η(α) is monotonically increasing, the solution to the constrained optimization problem in Equation (13) is the largest replacement ratio within the feasible set:

(13) α∗ = max(*A*) = 50%

The 50% replacement ratio is thus the value that maximizes the thermo-mechanical index η subject to the compressive-strength requirement for structural strengthening, rather than a value selected qualitatively after η was computed.

Taken together, the constrained optimization in Equation (13) provides a rigorous, criterion-based derivation of the 50% replacement ratio, while the saturation of thermal-conductivity improvement and the diminishing marginal gain in η provides an independent physical corroboration of this result. At 50% replacement, nearly the full achievable reduction in thermal conductivity is realized while more than 95% of the compressive strength is retained.

## 4. Thermal Modeling and Parametric Optimization of B-ECC Overlay Walls

### 4.1. Development and Validation of the Finite-Element Model

#### 4.1.1. Model Geometry and Boundary Conditions

A two-dimensional longitudinal-section finite-element model of the wall was developed. From exterior to interior, the model comprised an exterior air film represented by a boundary condition, a B-ECC strengthening layer of parameterized thickness, sintered porous-brick masonry of parameterized thickness, a second B-ECC strengthening layer, and an interior air film. The exterior surface was held at −20 °C, the interior surface at 18 °C, and the lateral boundaries were adiabatic.

#### 4.1.2. Mesh Discretization and Convergence Analysis

Four-node two-dimensional linear heat-transfer elements (D_C2_D_4_) were used. A convergence study showed that a medium mesh with a 5 mm element size was sufficient, with a relative error below 1%.

#### 4.1.3. Thermophysical Material Properties

Masonry (240 mm thick mortar-bonded fired clay brick): λ = 0.51 W/(m·K), c = 900 J/(kg·K), and ρ = 1800 kg/m^3^. B-ECC (50% glazed-hollow-bead replacement): λ = 0.244 W/(m·K), c = 1050 J/(kg·K), and ρ = 1550 kg/m^3^. Air films: Re = 0.05 m^2^·K/W and Ri = 0.15 m^2^·K/W.

### 4.2. Model Validation and Accuracy Assessment

**Figure 8:**
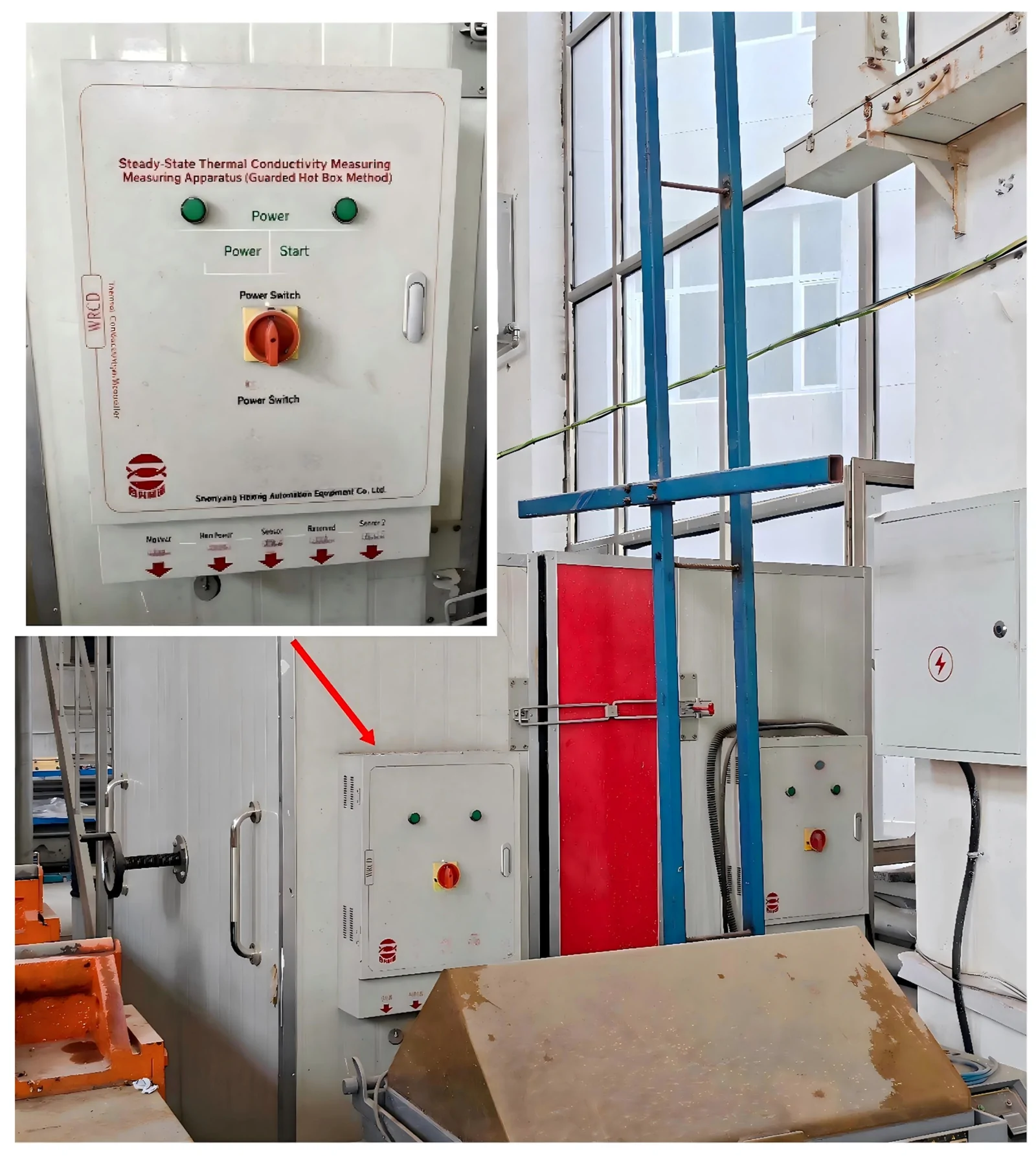
Thermal-conductivity measurement equipment.

To assess the predictive accuracy of the finite-element model described in Section 4.1, a steady-state thermal test was conducted on masonry wall specimens. The test equipment is shown in Figure 8. The following subsections detail the specimen configuration, instrumentation, testing procedure, and data reduction methodology to ensure the reproducibility of the validation experiment.

#### 4.2.1. Specimen Description

Three groups of wall specimens were prepared, as listed in Table 4. Each specimen measured 1.0 m (width) × 1.0 m (height). The masonry wythes were constructed from common fired-clay bricks (240 mm × 115 mm × 53 mm) and M1 mortar, with a thickness of 240 mm. For the strengthened specimens, a 20 mm or 30 mm layer of the B-ECC (50% glazed-hollow-bead replacement, 1.5% fiber content) was applied to both faces of the masonry using a plastering technique, replicating the intended field application. All specimens were cured under standard laboratory conditions (20 ± 2 °C, 95% relative humidity) for 28 days prior to testing.

#### 4.2.2. Test Setup and Instrumentation

The test was conducted using a guarded hot-box apparatus. The “cold side” of the apparatus was an insulated chamber in which the temperature was maintained at −15 ± 2 °C by circulating a coolant solution. The “hot side” consisted of an electric heating film controlled by a variable autotransformer to maintain a constant surface temperature of 18 ± 0.5 °C on the interior face of the specimen. The specimen was sealed into the opening between the two chambers with high-performance insulation to prevent edge heat losses.

Temperatures were measured using T-type thermocouples (copper-constantan, accuracy ±0.2 °C). A total of 15 thermocouples were arranged in five rows across the specimen’s surface. On each face (hot and cold), three thermocouples were attached directly to the specimen surface using thermally conductive adhesive tape to measure the surface temperature. An additional three thermocouples were embedded at the interface between the masonry and the B-ECC layer during specimen fabrication to capture the temperature gradient. Heat flux through the specimen was measured using three thin-film heat flux sensors (accuracy ± 3%) placed on the centerline of the hot-side surface.

#### 4.2.3. Test Procedure and Steady-State Criterion

After the specimen was installed and the chambers were sealed, the cooling system and the heating film were activated simultaneously. The test was monitored continuously. A steady-state condition was considered achieved when the following criteria were met simultaneously: the temperature fluctuation at all measurement points was less than ±0.3 °C over a period of 30 min and the variation in the measured heat flux was less than ±2% over the same 30 min period.

The test duration required to reach steady state was approximately 4 to 6 h for the unstrengthened specimen and 6 to 8 h for the B-ECC-strengthened specimens. Once steady-state conditions were confirmed, temperature and heat flux data were recorded at 1 min intervals for an additional 30 min.

#### 4.2.4. Determination of the U-Value and Measurement Uncertainty

The overall heat-transfer coefficient (U-value) was determined from the steady-state measurements as

(14) $$U=\frac{q_{\mathrm{avg}}}{T_{\mathrm{hot}}-T_{\mathrm{cold}}}$$

where *q*_avg_ was the average measured heat flux over the 30 min steady-state recording period.

*T*_hot_ and *T*_cold_ were the average surface temperatures of the specimen on the hot and cold sides, respectively.

The combined uncertainty in the measured U-value was estimated by considering the following sources: the accuracy of the heat flux sensors (±3%); accuracy of the thermocouples (±0.2 °C, contributing approximately ±1.0% to the U-value uncertainty); and heat loss at specimen edges (estimated at ±1.5% through pre-test calibration). The total combined relative uncertainty in the reported U-values is estimated to be approximately ±4.5%.

#### 4.2.5. Comparison of Experimental and Numerical Results

The experimental U-values were compared with the simulated results from the finite-element model. As shown in Table 4, the relative errors between the experimental and numerical results were −2.6%, −4.1%, and +2.1% for the three test cases, respectively. All deviations were within the acceptable accuracy range of ±5%, confirming that the finite-element model provided sufficient predictive capability for the subsequent parametric analysis. The minor discrepancies can be attributed to simplifications in the model’s assumed material homogeneity and the unavoidable measurement uncertainties in the experiment.

### 4.3. Parametric Sensitivity Analysis

#### 4.3.1. Effect of Masonry Thickness on the U-Value

The U-values of walls with different masonry thicknesses are compared in Table 5. The strengthening layers were fixed at δ_ECC_ = 20 mm on each face, the glazed-hollow-bead replacement ratio was 50%, and the winter temperature difference was 38 °C.

The marginal benefit of increasing wall thickness diminished. Increasing the thickness from 120 to 240 mm reduced the U-value by 27.6%; increasing it from 240 to 370 mm reduced the U-value by only 16.8%, and increasing it from 370 to 490 mm reduced the U-value by only 9.8%. The strengthening layer made its largest relative contribution to the 120 mm wall, accounting for 26.7% of the total thermal resistance.

#### 4.3.2. Effect of Strengthening-Layer Thickness on the U-Value

Table 6 compares the U-values obtained at different strengthening-layer thicknesses. The masonry thickness was fixed at 240 mm, representing a typical exterior wall, and the glazed-hollow-bead replacement ratio was 50%.

**Figure 9:**
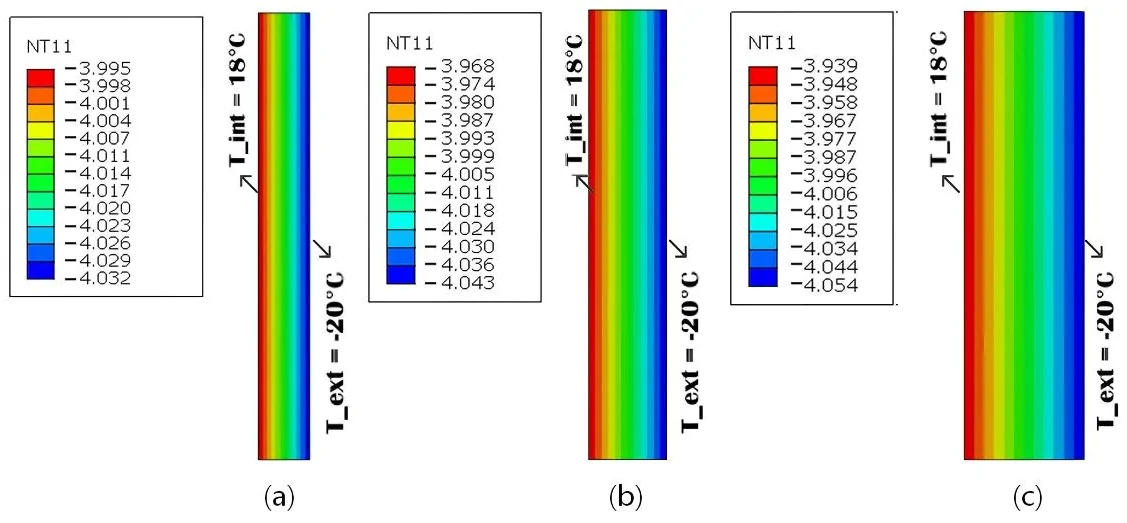
Thermal performance of masonry walls with different thicknesses: (**a**) heat-transfer contour for the 120 mm wall; (**b**) heat-transfer contour for the 240 mm wall; and (**c**) heat-transfer contour for the 370 mm wall.

**Figure 10:**
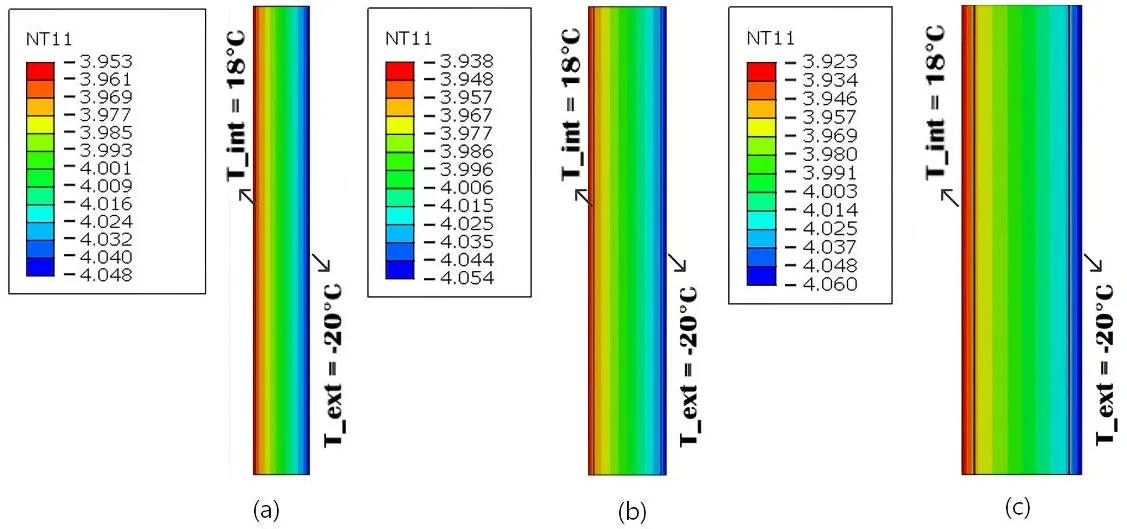
Thermal performance of walls with different strengthening-layer thicknesses: (**a**) heat-transfer contour for the 10 mm ECC layer; (**b**) heat-transfer contour for the 20 mm ECC layer; and (**c**) heat-transfer contour for the 30 mm ECC layer.

**Figure 11:**
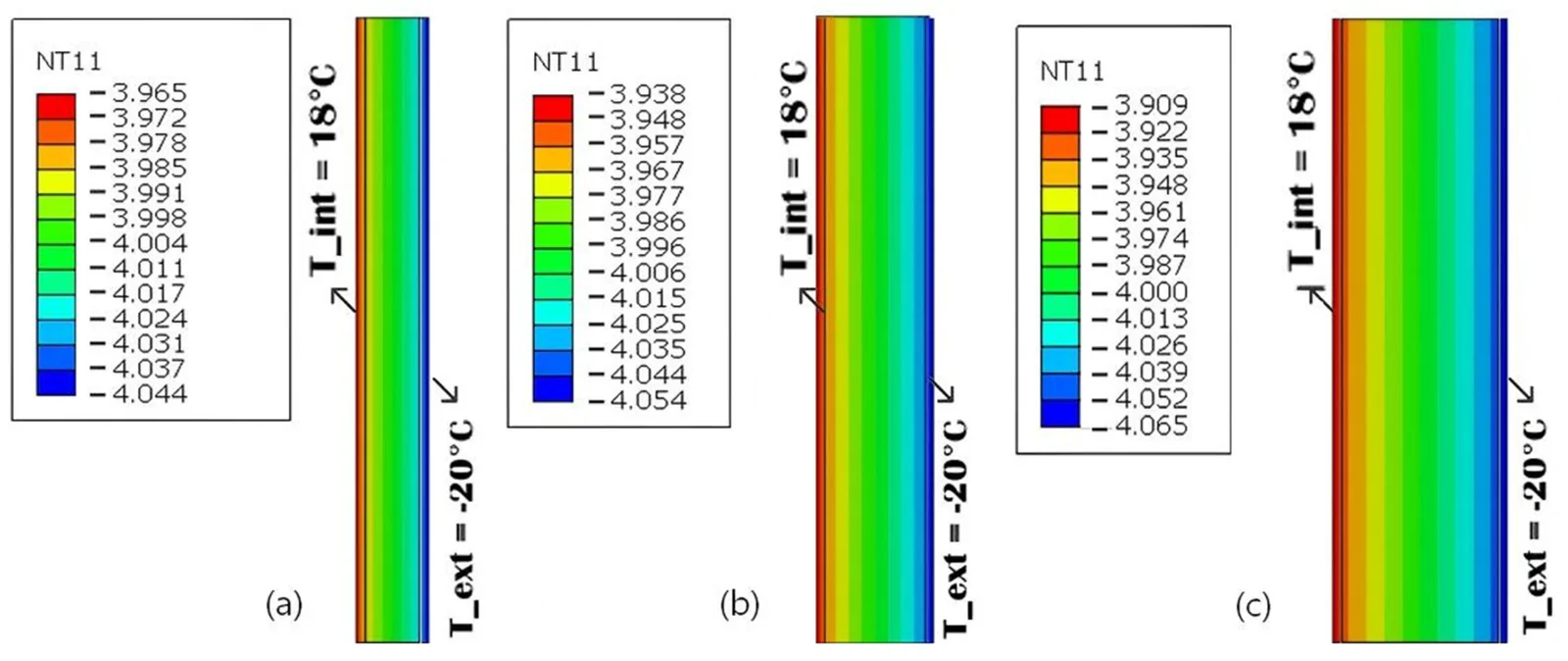
Comparison of the thermal performance of wall models: (**a**) heat-transfer contour for the 120 mm B-ECC overlay wall; (**b**) heat-transfer contour for the 240 mm B-ECC overlay wall; and (**c**) heat-transfer contour for the 370 mm B-ECC overlay wall.

The incremental insulation benefit was a diminishing function of layer thickness. Increasing the thickness from 10 to 20 mm added 9.3 percentage points of improvement, whereas increasing it from 20 to 30 mm added only 6.4 percentage points. Thermal performance continued to improve monotonically up to 30 mm, the maximum thickness examined, with no interior optimum identified within this range. A thickness of 20 mm is therefore recommended based on the diminishing marginal thermal benefit and general constructability considerations for cementitious overlays, rather than as a result of a formal multi-criteria optimization incorporating cost or structural requirements, as shown in Figure 9, Figure 10 and Figure 11.

#### 4.3.3. Multifactor Coupling Analysis

The coupled effects of masonry thickness (120, 240, 370, and 490 mm) and strengthening-layer thickness (0–30 mm) were evaluated. The U-values for all cases are summarized in Table 7.

Key finding: The 120 mm wall was most sensitive to strengthening, with a 33.7% reduction in U-value after application of 20 mm layers. The 240 mm wall provided the best overall balance, with a 27.3% reduction. For walls 370 mm or thicker, the thermal benefit was relatively limited (less than 20%), and the primary function of the B-ECC was structural strengthening rather than thermal insulation.

### 4.4. Thermal Performance of Different Wall Configurations

Four wall configurations were compared: (1) unstrengthened 240 mm masonry; (2) reinforced-concrete strengthening comprising 20 mm concrete + 240 mm masonry + 20 mm concrete; (3) mortar strengthening comprising 20 mm reinforced cement mortar + 240 mm masonry + 20 mm reinforced cement mortar; and (4) B-ECC strengthening comprising 20 mm B-ECC + 240 mm masonry + 20 mm B-ECC. The results are shown in Table 8.

**Figure 12:**
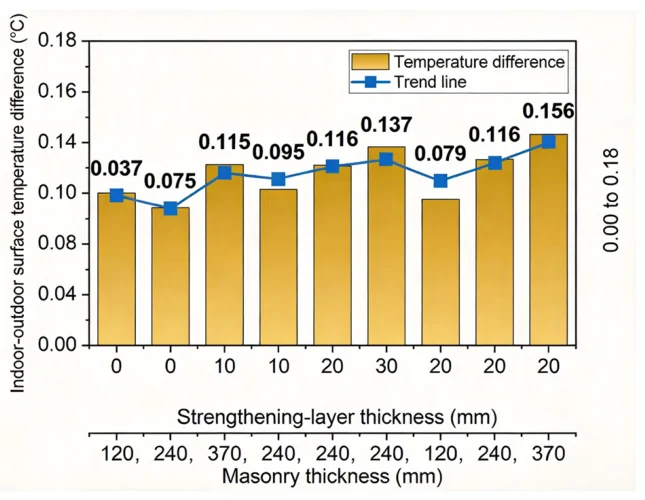
Comparison of indoor-to-outdoor surface temperature differences for walls with different parameters.

Although conventional reinforced-concrete strengthening provides high load-bearing capacity, its high thermal conductivity of 2.0 W/(m·K) actually increases heat loss. A B-ECC simultaneously improves structural and thermal performances and offers the best balance between thermal effectiveness and economy, as shown in Figure 12.

### 4.5. Applicability of the Thermal Model

#### 4.5.1. Quantitative Effects of Model Assumptions

The finite-element model employs the following simplified assumptions: the steady-state assumption (this model only calculates the daily average steady-state heat transfer; instantaneous heat flux variations induced by diurnal temperature fluctuations are not considered; an analysis of the impact of the daily average heat-transfer coefficient is provided in Section 2.3.1); neglect of thermal radiation (under ambient-temperature conditions, radiative heat transfer accounts for less than 5% of the total heat transfer); and a two-dimensional (2D) plane model (this approach has limited accuracy in simulating three-dimensional thermal bridges; however, this limitation is compensated for using a linear thermal bridge correction factor).

#### 4.5.2. Parameter Sensitivity

The effects of uncertainty in key parameters were as follows: a ±5% change in thermal conductivity changed the U-value by approximately ±3%; a ±10 mm change in strengthening-layer thickness changed the U-value by approximately ±8%; and uncertainty in surface-film resistance changed the U-value by approximately ±2%. The model was therefore most sensitive to thickness and least sensitive to surface-film resistance, consistent with engineering expectations.

## 5. Integrated Retrofit Design and Energy Assessment: Case Study

### 5.1. Project Background and Comparison Wall Definition

#### 5.1.1. Representativeness of the Case Building

**Figure 13:**
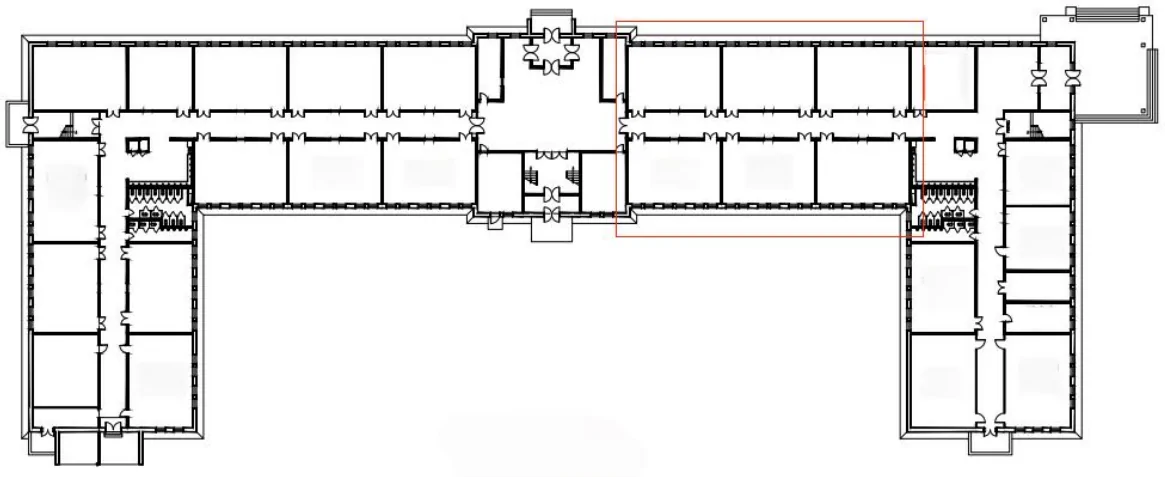
Structural plan of the first floor of the teaching building.

**Figure 14:**
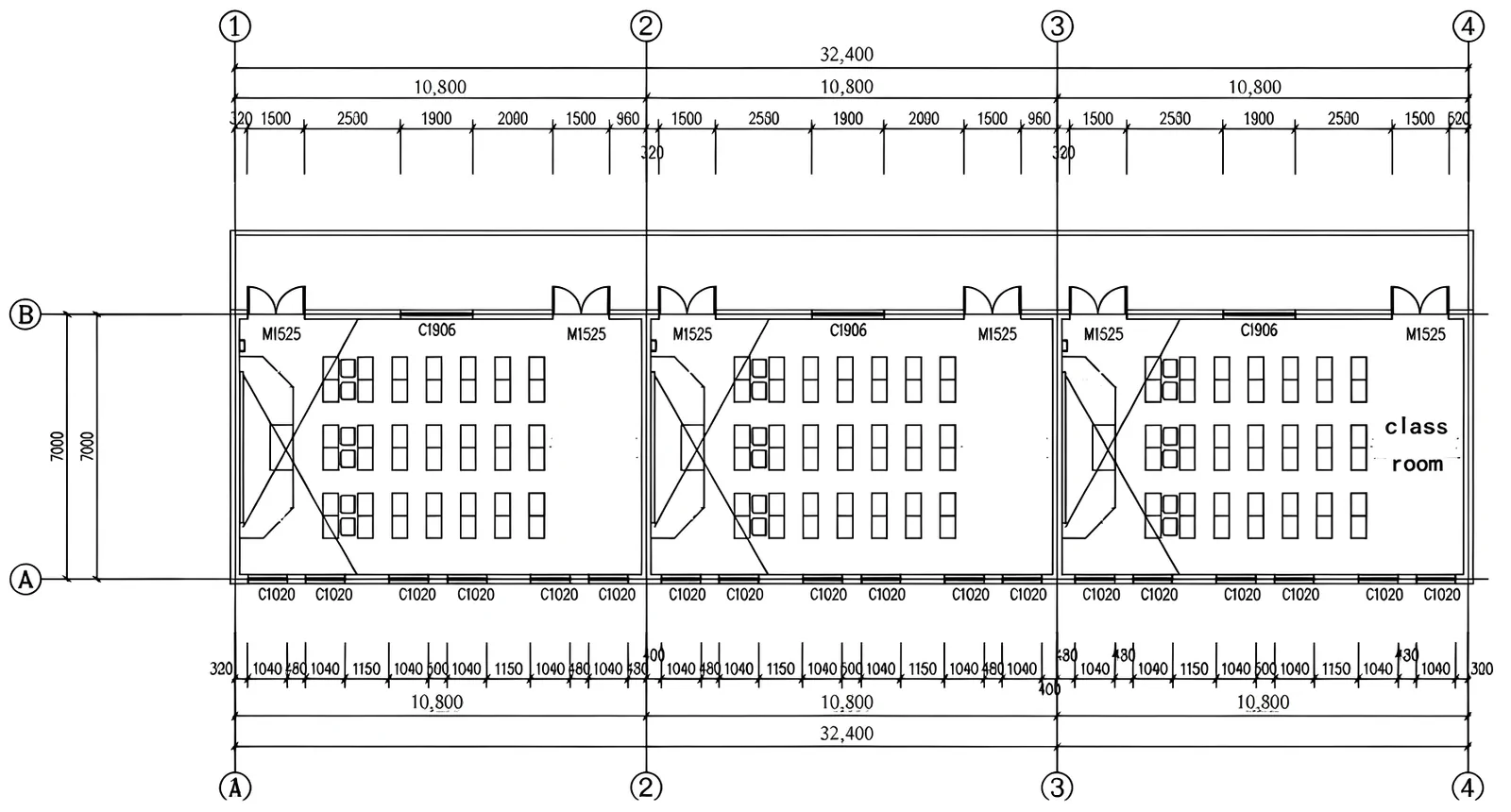
Surveyed plan of the selected teaching-building unit.

**Figure 15:**
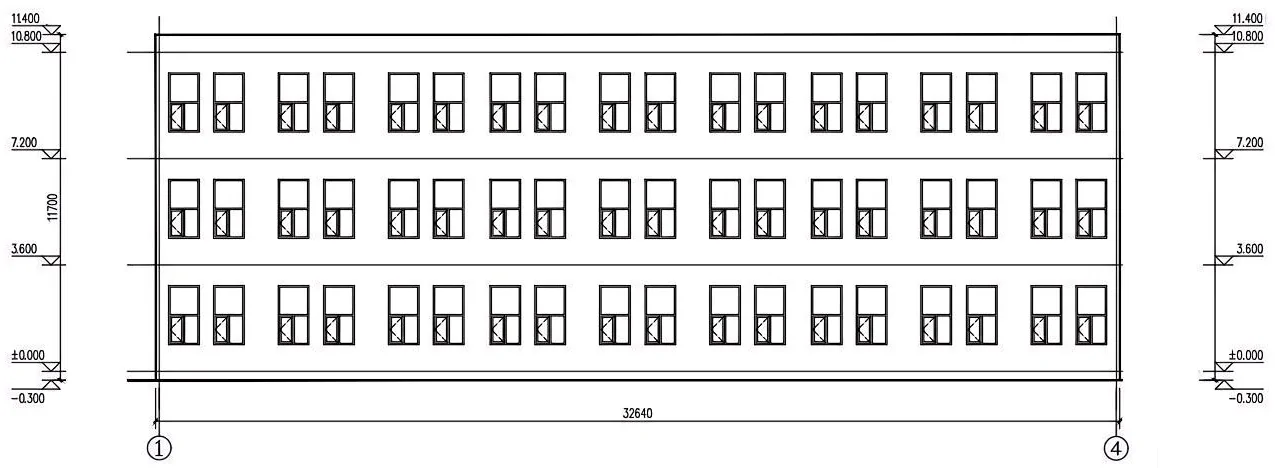
Surveyed south elevation in the selected teaching-building unit.

A representative three-story masonry teaching building was selected as the case study. Based on field-survey data, the building was constructed in the 1980s, has a rectangular plan, and lacks both ring beams and constructional columns as well as comprehensive seismic detailing, making it typical of aging masonry construction. The building is 10.88 m long and 7 m wide, with three stories of 3.6 m each. Its cast-in-place floor slabs are 100 mm thick. The exterior and interior walls are 490 and 240 mm thick, respectively. The structural plan is shown in Figure 5, and one unit between deformation joints was selected for analysis, as shown in Figure 13, Figure 14 and Figure 15. The exterior surface area is 1090 m^2^. The exterior walls are reported as 490 mm thick and the interior walls as 240 mm thick and consist of MU7.5 fired clay bricks laid in M1 mortar. The annual heating demand is approximately 70,685 kWh/a, with an annual heating cost of approximately RMB 21,000.

#### 5.1.2. Retrofit Scenarios and Benchmarking

Four retrofit scenarios were defined:

Scenario 1 (comparison wall): A 490 mm fired-clay-brick masonry, with no insulation, single-glazed steel windows, no roof insulation, Class II airtightness, and natural ventilation without heat recovery.

Scenario 2 (conventional strengthening): 20 mm reinforced mortar + 490 mm brick masonry, with no insulation, window replacement, or ventilation retrofit.

Scenario 3 (B-ECC strengthening): 20 mm B-ECC layers on both faces + 490 mm brick masonry, with no insulation, window replacement, or ventilation retrofit.

Scenario 4 (integrated retrofit): 20 mm B-ECC layers on both faces + 120 mm external graphite-expanded-polystyrene insulation + 490 mm brick masonry; an additional 140 mm XPS roof-insulation layer; triple-glazed, double-cavity, low-E insulating windows (U = 1.4 W/(m^2^·K)); and a mechanical ventilation system with 70% heat-recovery efficiency.

### 5.2. Calculation of Thermal Parameters for the Four Scenarios

#### 5.2.1. Thermal Performance of the Comparison Wall (Scenario 1)

Exterior-wall U-value: For the uninsulated brick masonry wall, U = 1/(0.05 + 0.961 + 0.15) = 0.861 W/(m^2^·K). Including the linear thermal-bridge term Ψ_1_L_1_ = 125.70 W/K gives a mean U-value Um = 0.861 + 125.70/1097.72 = 0.975 W/(m^2^·K).

Roof U-value: The cast-in-place concrete slab with a cinder screed had no effective insulation, giving Uroof = 4.57 W/(m^2^·K). Windows: The single tempered glazing had an overall U-value of approximately 4.7 W/(m^2^·K). Airtightness: *n* ≈ 1.0 h^−1^, as shown in Table 9.

The annual heating demand (BECS dynamic simulation): Qannual = 70,685 kWh/a. The area-normalized heating demand qH is 99.7 kWh/(m^2^·a), and the building heat-demand index is approximately 35 W/m^2^, 1.46 times the allowable value.

#### 5.2.2. Thermal Performance of the Conventional Strengthening Scheme (Scenario 2)

Exterior-wall assembly: 20 mm steel-mesh-reinforced cement mortar (λ = 1.74 W/(m·K)) + 490 mm fired-clay-brick masonry (λ = 0.51 W/(m·K)), strengthened on both faces.

Exterior-wall U-value: The clear-wall value is U = 1/(0.15 + 0.972 + 0.05) = 0.89 W/(m^2^·K). Including the linear thermal-bridge term Ψ_1_L_1_ = 124.28 W/K gives a mean value Um = 0.89 + 124.28/660.96 = 1.08 W/(m^2^·K).

Roof U-value: As in Scenario 1, with no insulation, Uroof ≈ 4.57 W/(m^2^·K). Windows: As in Scenario 1, with a single tempered glazing, U ≈ 4.7 W/(m^2^·K). Airtightness: *n* ≈ 1.0 h^−1^, as shown in Table 10.

The annual heating demand (dynamic simulation): Qannual = 70,118 kWh/a, and qH = 98.9 kWh/(m^2^·a). The energy-saving rate is η = (70,685 − 70,118)/70,685 = 0.8%, indicating almost no energy-saving benefit.

#### 5.2.3. Thermal Performance of B-ECC Strengthening (Scenario 3)

Exterior-wall assembly: 20 mm B-ECC strengthening layers on both faces (λ = 0.244 W/(m·K)) + 490 mm fired-clay-brick masonry (λ = 0.51 W/(m·K)).

Exterior-wall U-value: The clear-wall value is U = 1/(0.15 + 1.043 + 0.05) = 0.74 W/(m^2^·K). Including the linear thermal-bridge term Ψ_1_L_1_ = 106.26 W/K gives a mean value of Um = 0.74 + 106.26/660.96 = 0.91 W/(m^2^·K).

Roof U-value: As in Scenario 1, Uroof ≈ 4.57 W/(m^2^·K). Windows: As in Scenario 1, U ≈ 4.7 W/(m^2^·K). Airtightness: n ≈ 1.0 h^−1^, as shown in Table 11.

The annual heating demand (dynamic simulation): Qannual = 65,795 kWh/a, and q_H_ = 92.8 kWh/(m^2^·a). The energy-saving rate is η = (70,685 − 65,795)/70,685 = 6.9%. Thermal performance improved by approximately 16% relative to the comparison wall, but remained far from the 75% energy-saving target.

#### 5.2.4. Thermal Parameters of the Integrated Retrofit (Scenario 4)

Retrofitted exterior-wall assembly, from exterior to interior: A 5 mm crack-resistant mortar with alkali-resistant mesh; a 120 mm graphite-expanded-polystyrene (SEPS) board [λ = 0.032 × 1.20 = 0.0384 W/(m·K)]; a 20 mm B-ECC strengthening layer [λ = 0.244 W/(m·K)]; a 490 mm fired-clay-brick masonry [λ = 0.51 W/(m·K)]; and a 20 mm interior B-ECC strengthening layer, as shown in Table 12.

R0 = 0.15 + 4.255 + 0.05 = 4.455 m^2^·K/W and U = 1/4.455 = 0.224 W/(m^2^·K). After insulation was added, the thermal-bridge term decreased to 30.10 W/K, a reduction of 76.1%, and Um = 0.224 + 30.10/1097.72 = 0.251 W/(m^2^·K).

Thermal-resistance breakdown before and after retrofit: Before retrofit, the total exterior-wall thermal resistance was only 1.161 m^2^·K/W, of which masonry contributed 83% and the interior and exterior surface films contributed 17%. After retrofit, the total resistance increased to 4.455 m^2^·K/W. The insulation board contributed 3.125 m^2^·K/W (70.1%), the two B-ECC layers 0.164 m^2^·K/W (3.7%), the masonry 0.961 m^2^·K/W (21.6%), and the surface films 4.5%. The insulation board was therefore the dominant source of the increase in thermal resistance, whereas the direct thermal contribution of the B-ECC layers was relatively modest (3.7% of total resistance). The primary functions of the B-ECC in the integrated system are structural and interfacial, as discussed in Section 5.3.

Other retrofit measures: After roof retrofit, Uroof = 0.229 W/(m^2^·K). After window replacement, U = 1.4 W/(m^2^·K). The heat-recovery efficiency of the ventilation system was 70%, corresponding to a correction factor of 0.30, as shown in Table 13.

The annual heating demand (BECS dynamic simulation): Qannual, retrofit = 17,229 kWh/a and qH = 24.3 kWh/(m^2^·a). The energy-saving rate is η = (70,685 − 17,229)/70,685 = 75.6%, meeting and exceeding the 75% target, as shown in Table 14.

### 5.3. Synergistic Energy-Saving Mechanisms and Contributions of Multiple Measures

The contributions were ranked as follows: window replacement (25.7%) > wall insulation system comprising B-ECC + SEPS (22.0%) > roof insulation (11.7%) > heat-recovery ventilation (8.3%) > B-ECC strengthening alone (6.0%) > perimeter floor insulation (1.9%), as shown in Table 15.

1. Window replacement was the highest-priority measure. Although windows represented only 17% of the envelope area, the U-value of the existing single glazing was as high as 4.7 W/(m^2^·K), causing five to eight times the heat loss per unit area of the exterior wall.
2. The wall insulation system ranked second. B-ECC strengthening alone contributed 6.0% to the overall energy-saving rate, primarily through the reduction in thermal bridging and the additional thermal resistance of the B-ECC layers themselves. When combined with 120 mm of SEPS external insulation, the wall system as a whole contributed 22.0%.

It should be noted that the contribution of each measure reported in Table 16 was determined by sequentially adding measures in a fixed order (B-ECC layers → wall insulation → roof insulation → window replacement → floor insulation → ventilation). Under this method, the contribution assigned to each measure depends on the order of introduction, because the thermal and ventilation effects are not strictly additive. For example, if window replacement were introduced before wall insulation, the relative share of each measure would shift. The sequential addition method therefore provides an indicative ranking of retrofit priorities rather than an exact quantification of each measure’s independent contribution. Nevertheless, the ranking obtained—window replacement > wall insulation system > roof insulation > heat-recovery ventilation > B-ECC strengthening—is consistent with engineering experience in severe-cold regions and with findings from existing retrofit studies, suggesting that the overall priority order is robust.

### 5.4. Economic and Environmental Assessment of the Retrofit

#### 5.4.1. Estimated Retrofit Investment

The incremental investment per unit floor area was RMB 521,500/709 m^2^ = RMB 736/m^2^. Because strengthening and energy retrofit were implemented concurrently, scaffolding and temporary facilities were shared, avoiding approximately 12–15% of duplicated expenditure. The resulting net incremental investment was approximately RMB 443,000–459,000, equivalent to RMB 625–648/m^2^, as shown in Table 16.

#### 5.4.2. Economic Benefits

The annual heating-cost savings: ΔQ = 53,456 kWh/a. At the Changchun district-heating tariff of RMB 30/(m^2^·a), the annual saving was RMB 16,100.

The simple payback period was 52.15/1.61 = 32.4 years based on the total incremental investment. The discounted payback period at a 5% discount rate was approximately 42 years. The net present value over a 30-year service life at a 5% discount rate was RMB −62,000. From heating-cost savings alone, the economic performance is therefore weak, and the retrofit cannot be justified on energy-cost grounds alone. This limitation should be weighed against the non-energy benefits discussed below. Nevertheless, this type of retrofit is generally a government-funded decarbonization program for existing school buildings, and its comprehensive assessment should also include improved comfort and indoor environmental quality.

#### 5.4.3. Environmental Benefits and Carbon Reduction

The annual heating-energy saving is equivalent to 8.23 t of standard coal. Based on the emission factors for district heating in the Changchun severe-cold region (which relies on coal-fired combined heat and power plants)—2.625 t CO_2_/tce, 0.255 t SO_2_/tce, and 0.182 t particulates/tce, derived from the National Energy Administration’s default emission coefficients—the annual reductions are 21.6 t of CO_2_, 2.1 t of SO_2_, and 1.5 t of particulates. The corresponding 30-year operational CO_2_ reduction is 648 t, equivalent to the annual emissions avoided by 133 electric vehicles each traveling 25,000 km. It should be noted that this figure represents operational emission reductions only and does not account for embodied emissions from the production of the B-ECC, EPS/XPS insulation, new windows, ventilation equipment, construction activities, maintenance, or end-of-life disposal. A full life-cycle carbon assessment would be required to quantify the total net emission reduction.

#### 5.4.4. Overall Benefit Assessment

Table 17 compares thermal performance and comfort before and after retrofit. For thermal performance, the exterior-wall U-value decreased from 0.975 to 0.251 W/(m^2^·K), a reduction of 74.3%; the roof U-value decreased from 4.57 to 0.229 W/(m^2^·K), a reduction of 95.0%; and the mean U-value decreased from 1.09 to 0.28 W/(m^2^·K). For comfort, the winter indoor temperature fluctuation decreased from ±3 °C to ±0.5 °C, and indoor air quality improved from uncontrolled cold-air infiltration to heat-recovery ventilation with an air-change rate greater than 3 h^−1^. The retrofit thus reduced envelope heat loss and improved quantified indoor-environmental conditions.

### 5.5. Discussion

The key results of this study are discussed below in the context of previous experimental and numerical studies.

Regarding thermal conductivity, the B-ECC with 50% glazed-hollow-bead replacement achieved λ = 0.244 W/(m·K), which was substantially lower than that of the conventional ECC (0.7–0.9 W/(m·K) reported by Shah et al. [10]) and of lightweight-aggregate concrete (0.4–0.6 W/(m·K) reported by Neville [13]). It is also noticeably lower than the 0.478–0.668 W/(m·K) range reported for a carbon-fiber-reinforced ultra-lightweight ECC (ULW-ECC) with fly-ash cenospheres [44] and comparable to the lower bound of 0.20–0.73 W/(m·K) achieved by an air-entrained ULW-ECC [45]. This confirms that glazed hollow beads, with their high closed-cell ratio (93%), are an effective lightweight aggregate for reducing ECC thermal conductivity.

In terms of mechanical performance, the B-ECC retained a 28-day compressive strength of 43.9 MPa at 50% replacement, corresponding to a strength loss of only 4.8%. This is more favorable than the 27–32% strength reduction typically observed in rubberized ECC at comparable replacement levels [46] and demonstrates that the closed-cell structure of glazed hollow beads induces less mechanical penalty than open-pore lightweight aggregates.

For the building-level energy assessment, the integrated retrofit achieved a 75.6% energy-saving rate and an exterior-wall U-value of 0.251 W/(m^2^·K). These results are consistent with the 60–75% energy-saving range reported for comprehensive envelope retrofits of existing masonry buildings in severe-cold regions of China [23,27], confirming that the proposed integrated strategy meets the national energy-efficiency target.

Overall, the B-ECC thermal conductivity and strength results align with the lower end of the ranges reported for other modified ECC systems, while the building-level energy performance is consistent with established retrofit benchmarks in the same climate zone.

## 6. Conclusions and Outlook

This study developed a glazed-hollow-bead-modified engineered cementitious composite (B-ECC) and systematically evaluated its thermal performance across three scales: material, wall component, and whole building. The following conclusions are drawn:

1. A B-ECC provides balanced thermo-mechanical performance. At 50% glazed-hollow-bead replacement and 1.5% fiber content, thermal conductivity reached 0.244 W/(m·K) (a 52% reduction) while 28-day compressive strength remained 43.9 MPa (only 4.8% loss), meeting masonry strengthening requirements.
2. A 20 mm overlay per face is recommended. Simulations, validated by guarded hot-box experiments (±5% error), showed that increasing overlay thickness from 0 to 20 mm improved insulation by 20.0%, whereas a further increase to 30 mm added only 6.4%, indicating diminishing marginal returns.
3. The integrated retrofit achieved a 75.6% energy-saving rate. For a representative teaching building in Changchun, combining B-ECC overlays with external insulation, roof insulation, window replacement, and heat-recovery ventilation reduced the annual heating demand from 70,685 to 17,229 kWh/a. The exterior-wall U-value decreased from 0.975 to 0.251 W/(m^2^·K).
4. Contribution ranking of measures: Window replacement (25.7%) > wall insulation system (22.0%) > roof insulation (11.7%) > heat-recovery ventilation (8.3%) > B-ECC alone (6.0%). This ranking is valid only for the specific sequential implementation order adopted in this study, because the sequential addition method cannot prove mutual independence among individual retrofit measures. No single measure could independently achieve the 75% target. The B-ECC’s primary role is thermal-bridge mitigation rather than direct insulation.
5. Environmental and economic assessment: The retrofit yields an estimated 648 t of CO_2_ reduction over 30 years, with a simple payback period of 32.4 years based on heating-cost savings alone. When improved indoor thermal comfort and better indoor air quality are considered, the retrofit becomes justifiable within government-funded decarbonization and school-safety programs.
6. Limitations: The economic assessment excludes carbon trading revenues and secondary benefits. The environmental assessment is limited to operational emissions. Structural performance enhancement requires further experimental validation. Future work should address a life-cycle carbon analysis and the seismic testing of B-ECC overlay walls.

## Figures and Tables

**Table 1:** Properties of the glazed hollow beads.

Property	Bulk Density (kg/m^3^)	Cylinder Compressive Strength (kPa)	Thermal Conductivity [W/(m·K)]	Volumetric Water Absorption (%)	Closed-Cell Ratio (%)
Measured value	110	196	0.042	35	93
Standard requirement	80–120	≥150	≤0.048	≤45	≥80

**Table 2:** Properties of the PVA fibers.

Material	Length (mm)	Diameter (mm)	Density (g/cm^3^)	Elastic Modulus (GPa)	Ultimate Tensile Strength (MPa)
PVA fibers	16	0.04	1.3	40	1600

**Table 3:** Reference mixture proportions (1.5% fiber content and 50% glazed-hollow-bead replacement).

Constituent	Cement	Fly Ash	Quartz Sand	Glazed Hollow Beads	Water	Superplasticizer	PVA Fibers
Dosage (kg/m^3^)	585	715	338.5	32.95	365	3–5	19.5

**Table 4:** Comparison of experimental and simulated results.

Case	Experimental U-Value, Uexp	Simulated U-Value, Usim	Relative Error
240 mm masonry + 20 mm B-ECC	0.878	0.855	−2.6%
240 mm masonry + 30 mm B-ECC	0.773	0.741	−4.1%
240 mm masonry (unstrengthened)	1.152	1.176	+2.1%

**Table 5:** U-values for different masonry thicknesses.

Masonry Thickness (mm)	Masonry Thermal Resistance	Strengthening-Layer Thermal Resistance	Total Thermal Resistance	U-Value [W/(m^2^·K)]	Reduction Relative to Comparison Wall
120	0.235	0.164	0.614	1.63	Comparison wall
240	0.471	0.164	0.850	1.18	−27.6%
370	0.725	0.164	1.104	0.906	−44.4%
490	0.961	0.164	1.340	0.746	−54.2%

**Table 6:** U-values for different strengthening-layer thicknesses.

Strengthening-Layer Thickness (mm)	Strengthening-Layer Thermal Resistance	Total Thermal Resistance	U-Value [W/(m^2^·K)]	Relative Improvement in Insulation Performance
0 (unstrengthened)	0	0.686	1.458	—
10	0.082	0.768	1.302	10.7%
20	0.164	0.850	1.176	20.0%
30	0.246	0.932	1.073	26.4%

**Table 7:** U-values for all parametric cases.

Masonry Thickness (mm)	*δ*_ECC_ = 0 mm	10 mm	20 mm	30 mm
120	1.63	1.45	1.30	1.22
240	1.18	1.11	0.99	0.91
370	0.91	0.86	0.77	0.72
490	0.75	0.71	0.64	0.59

**Table 8:** Comparison of thermal performance.

Alternative	U-Value [W/(m^2^·K)]	Thermal Conductivity [W/(m·K)]	Thermal Inertia, D	Thermal Improvement
Unstrengthened	1.17	0.51	9.6	—
Concrete strengthening	1.24	2.0	9.8	No (6% increase)
Mortar strengthening	1.08	1.74	9.5	—
B-ECC strengthening	0.91	0.244	9.6	Yes (+22.2%)

**Table 9:** Heat−loss coefficients per unit temperature difference (Scenario 1).

Component	Area (m^2^)	U−Value [W/(m^2^·K)]	Heat Loss (W/K)	Share (%)
Exterior walls	660.96	0.975	644.4	21.5
Roof	228.00	4.57	1042.0	34.7
Windows	190.00	4.70	893.0	29.7
Doors	12.00	4.70	56.4	1.9
Infiltration	—	—	366.1	12.2
Total	1090	—	3001.9	100.0

**Table 10:** Heat-loss coefficients per unit temperature difference (Scenario 2).

Component	Area (m^2^)	U-Value [W/(m^2^·K)]	Heat Loss (W/K)	Share (%)
Exterior walls	660.96	1.08	713.8	23.8
Roof	228.00	4.57	1042.0	34.7
Windows	190.00	4.70	893.0	29.7
Doors	12.00	4.70	56.4	1.9
Infiltration	—	—	366.1	12.2
Total	—	—	3071.3	100.0

**Table 11:** Heat-loss coefficients per unit temperature difference (Scenario 3).

Component	Area (m^2^)	U-Value [W/(m^2^·K)]	Heat Loss (W/K)	Share (%)
Exterior walls	660.96	0.91	601.5	20.1
Roof	228.00	4.57	1042.0	34.8
Windows	190.00	4.70	893.0	29.8
Doors	12.00	4.70	56.4	1.9
Infiltration	—	—	366.1	12.2
Total	—	—	2959.0	100.0

**Table 12:** Total thermal resistance of the exterior wall.

Layer	Thickness (mm)	λ [W/(m·K)]	Thermal Resistance (m^2^·K/W)
Crack-resistant mortar + mesh	5	0.930	0.005
Graphite EPS board (SEPS)	120	0.0384	3.125
B-ECC (exterior layer)	20	0.244	0.082
Fired-clay-brick masonry	490	0.51	0.961
B-ECC (interior layer)	20	0.244	0.082
Sum of material layers	655	—	4.255

**Table 13:** Heat-loss coefficients per unit temperature difference after retrofit.

Component	Area (m^2^)	U-Value [W/(m^2^·K)]	Heat Loss (W/K)	Share (%)	Reduction Relative to Scenario 1 (%)
Exterior walls	660.96	0.251	165.9	23.1	74.3
Roof	228.00	0.229	52.2	7.3	95.0
Windows	190.00	1.40	266.0	37.1	70.2
Doors	12.00	1.80	21.6	3.0	61.7
Infiltration + ventilation	—	—	209.2	29.2	75.7
Total	—	—	715.9	100.0	76.2

**Table 14:** Comparison of heating energy use among the four scenarios.

Scenario	Heating-Demand Index qH (W/m^2^)	Annual Heating Energy Use (kWh/a)	Energy Use per Unit Area [kWh/(m^2^·a)]	Energy-Saving Rate
Comparison wall	24.6	70,685	99.7	—
Steel-mesh mortar strengthening	24.4	70,118	98.9	0.8%
B-ECC strengthening	22.9	65,795	92.8	6.9%
Integrated retrofit	6.0	17,229	24.3	75.6%

**Table 15:** Analyzes of the contributions of each measure to the energy-saving target using sequential addition.

Cumulative Measures	Heat-Loss Coefficient per Unit Temperature Difference (W/K)	Cumulative Energy-Saving Rate (%)	Contribution of the Added Measure (%)
Scenario 1: Comparison wall	3001.9	0	—
+20 mm B-ECC layers on both faces	2822.1	6.0	6.0
+120 mm SEPS insulation + thermal-bridge treatment	2160.2	28.0	22.0
+140 mm XPS roof insulation	1810.2	39.7	11.7
+window replacement	1038.9	65.4	25.7
+perimeter floor insulation	981.4	67.3	1.9
+improved airtightness + heat-recovery ventilation	715.9	75.6	8.3

**Table 16:** Incremental investment for Scenario 4, including B-ECC strengthening.

Item	Quantity	Unit Price	Cost (RMB 10,000)
B-ECC strengthening layers on both faces (materials and installation)	660.96 m^2^	RMB 120/m^2^	7.93
External-wall insulation system (including finish and thermal-bridge treatment)	660.96 m^2^	RMB 185/m^2^	12.23
Roof insulation and waterproofing	228 m^2^	RMB 165/m^2^	3.76
Window replacement (triple glazing with two cavities and low-E coating)	190 m^2^	RMB 950/m^2^	18.05
Exterior doors and entrance vestibule	12 m^2^	RMB 1200/m^2^	1.44
Perimeter floor insulation	78 m^2^	RMB 120/m^2^	0.94
Heat-recovery ventilation system and airtightness treatment	709 m^2^	RMB 110/m^2^	7.80
Total	—	—	52.15

**Table 17:** Comparison before and after retrofit.

Indicator	Before Retrofit	After Retrofit	Improvement
**Thermal Performance**			
Exterior-wall U-value [W/(m^2^·K)]	0.975	0.251	74.3%
Roof U-value	4.57	0.229	95.0%
Mean U-value	1.09	0.28	74.3%
Comfort			
Indoor temperature fluctuation	±3 °C (winter)	±0.5 °C	Sixfold improvement in stability
Indoor air quality	Poor (cold-air infiltration)	Good (heat-recovery ventilation)	Air-change rate > 3 h^−1^

## Data Availability

The original contributions presented in this study are included in the article. Further inquiries can be directed to the corresponding author.

## Abbreviations

The following abbreviations are used in this manuscript:

ECC	Engineered cementitious composite
B-ECC	Glazed-Hollow-Bead-Modified ECC
